# Biology of the coconut bug, *Pseudotheraptus wayi* , on French beans

**DOI:** 10.1093/jis/14.1.44

**Published:** 2014-01-01

**Authors:** James Peter Egonyu, Sunday Ekesi, Jacques Kabaru, Lucy Irungu

**Affiliations:** 1 International Centre of Insect Physiology and Ecology-African Insect Science for Food and Health, P.O. Box 30772, 00100 Nairobi, Kenya; 2 School of Biological Sciences, University of Nairobi, P.O. Box 30197, 00100 Nairobi, Kenya

**Keywords:** Coreidae, development, fecundity, rearing, survival

## Abstract

The coconut bug, *Pseudotheraptus wayi* Brown (Heteroptera: Coreidae), is a major pest of a wide range of economically important crops in Eastern and Southern Africa. The suitability of French beans, *Phaseolus vulgaris* L. (Fabales: Fabaceae) as an alternative food for mass rearing of *P. wayi* was determined by elucidating its development, survival, and reproduction on French bean pods in the laboratory. Development and survival of immatures on French beans was comparable to what is reported with two hosts previously used for rearing this species, namely coconut and cashew. Adults survived thrice longer and laid almost twice more eggs on the French beans than was reported for the two hosts above. These findings suggest that French beans are more suitable for mass rearing of this species than coconut and cashew, which have been used previously but can be scarce and too costly.

## Introduction

The coconut bug, *Pseudotheraptus wayi* Brown (Heteroptera: Coreidae), is a major pest of a wide range of economically important crops in Eastern and Southern Africa, such as cashew ( *Anacardium occidentale* ), coconut ( *Cocos nucifera* ), macadamia ( *Macadamia integrifolia* ), carambola ( *Averrhoa carambola* ), pecan ( *Carya illinoinensis* ), cin-namon ( *Cinnamomum verum* ), loquat ( *Eriobotrya japonica* ), mango ( *Mangifera indica* ), avocado ( *Persea americana* ), guava ( *Psidium guajava* ), and cocoa ( *Theobroma cacao* ) ( Martin et al. 1997 ; Mitchell 2000 ; CABI 2005 ; Hill 2008 ; Maniania 2009 ; Nyambo 2009 ). Damage of up to 99.8% on coconut ( Way 1953 ), 52.4% on guava ( Van Der Meulen 1992 ), 76.2% on avocado fruits ( Van Der Meulen and Schoeman 1994 ), and 80% on cashew nut ( Nyambo 2009 ) has been attributed to this pest. Both nymphs and adults feed on the host plant, causing wilting and ne-crosis of young stems, leaves, inflorescences, and fruits as they suck sap and inject toxins into the host tissues ( Mitchell 2000 ; Hill 2008). Development of *P. wayi* from egg through five instars to the adult stage on coconut and cashew takes 31–41 days under different temperature regimes, while adult females survive on coconut for 45–66 days and males for 83–84 days, at 24.6°C ( Way 1953 ; Wheatley 1961 ; Mitchell 2000 ; CABI 2005 ). Preoviposition period on these hosts is 9–13 days, and eggs are laid singly at a rate of 2–3 per day, for a total lifetime fecundity of 74– 100 eggs per female ( Way 1953 ; Mitchell 2000 ).

A major focus of integrated pest management programs against this pest has been on the use of the predatory weaver ant, *Oecophylla longinoda,* which faces challenges such as its elimination by broad spectrum insecticides and competition and predation by other ants e.g., *Pheidole megacephala, P. punctulata, Anoplolepsis custodiens* . and *A. longipes* ( Martin et al. 1997 ; Mitchell 2000 ; Nyambo et al. 2003 ; CABI 2005 ). Effective management of *P. wayi* currently relies on application of insecticides such as cypermethrin, lambda-cyhalothrin, and endosulfan ( Martin et al. 1997 ; Mitchell 2000 ; CABI 2005 ), but these chemicals can be very hazardous to human and environmental health and are difficult to apply on tall trees. Environmentally sound management options for this pest are therefore still inadequate. Studies geared towards identification of effective and environmentally-friendly control options against the pest are essential but require an efficient procedure for mass rearing of insects of pre-determined reproductive stages and age. However, rearing materials such as coconut and cashew fruits may not be readily available and can be too costly ( Wheatley 1961 ). An alternative food substrate for mass rearing of *P. wayi* is therefore required. Here, we report the biology of *P. wayi* on French bean pods, *Phaseolus vulgaris* L. (Fabales: Fabaceae), which we selected for testing as an alternative food for the insects because this pest also attacks wild legumes ( Hill 2008 ).

## Materials and Methods

### Insect rearing

A stock culture of *P. wayi* originated from adults collected in June 2010 from cashew trees at the Kenya Agricultural Research Institute, Mtwapa Research Centre (3° 55’ S, 39° 44’ E, 15 m a.s.l.), Kilifi County, Kenya. The colony was reared in a laboratory maintained at 24.6 ± 1°C, 80 ± 1.3% RH, and photoperiod of 12:12 L:D at the International Centre of Insect Physiology and Ecology, Nairobi, Kenya.

**Figure 1.**
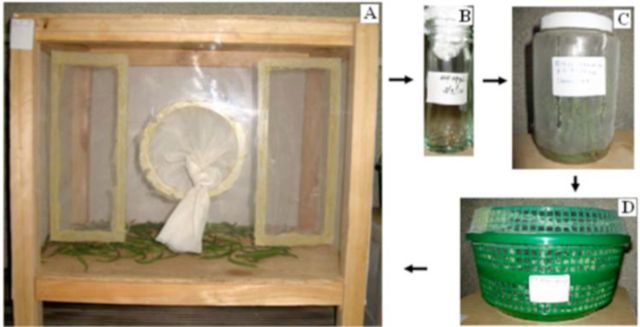
A pictorial summary of laboratory mass rearing of *Pseudotheraptus wayi* on French bean pods. **A:** an adult rearing cotton drill cage; **B:** an egg incubation glass vial; **C:** a plastic bottle for rearing nymphs for the first 3–4 days; **D:** a plastic basket for rearing nymphs until adult emergence. High quality figures are available online.

Figure 1 shows images of the cages used in rearing the insects. Adults were reared in wooden-cotton drill cages (61 x 46 x 46 cm), and the eggs were incubated in glass vials (2.5 cm inner diameter x 7.5 cm high) according to Wheatley (1961) . An attempt to rear nymphs in the wooden-cotton drill cage (23 x 30 x 30 cm) designed by Wheatley (1961) was unsuccessful because most of the newly hatched first instars were unable to timely detect the food and initiate feeding, probably due to excessive space in the cage, and starved to death. These cages were also inessential for the nymphs, unlike adults, which preferred to lay eggs on the cotton, therefore a relatively cheaper cage could suffice. To avoid these challenges, all first instars were transferred from the glass vials into small cylindrical plastic bottles (3.5 cm inner diameter x 6 cm high) using a camel-hair brush within 0–12 hr of hatching. The emerging nymphs had less space to wander around and were maintained in these bottles for 3–4 days. At this age, most nymphs had acclimatized to the food substrate and could search for it even in relatively more spacious cages. They were therefore transferred to a ventilated plastic basket (24 cm inner diameter x 16 cm high) lined at the bottom with filter paper (24 cm diameter) that was replaced weekly. The nymphs were maintained in these plastic baskets until the emergence of adults, which were transferred into the cotton drill cages described above.

All the rearing cages accommodated up to 50 individuals of the respective stages. In all cases, nymphs and adults fed *ad libitum* on fresh French bean pods, which were replaced twice a week. The insects were maintained for one generation before commencement of studies on the biology.

### Development and survival of immatures

A cohort of 100 eggs (0–2 hr old) was obtained from the colony described above and monitored daily for two weeks for hatching in glass vials, as described above. Similarly, a cohort of 100 newly emerged first instars (0– 12 hr old) was obtained and reared singly in the ventilated plastic bottles described above until adult emergence. Each nymph was monitored daily for molting (confirmed by the presence of exuviae, which were carefully removed using a camel hair brush) to the successive stages.

### Fecundity and longevity of adult stages

Ten pairs (female and male) of newly emerged (0–12 hr old) adults were each reared until death in wooden-cotton drill cages measuring 30 x 30 x 30 cm. Sexing was based on the characteristic sculptured lateral process on each side of the 9 ^th^ abdominal segment, which is only present in males ( Brown 1955 ). The eggs laid by each female from the test couples were enumerated and removed from the cages daily. Preoviposition period (number of days from adult female emergence to first egg laying), oviposition period (number of days from first to last egg laying), and postoviposition period (number of days from the last egg laying to the day of death) were computed.

### Data analysis

Means and standard errors of the means (SEM) were computed for the durations of the various life stages, fecundity (number of eggs laid by each female in its lifetime), and mean daily egg production (eggs per female per day). Survival of immatures (percentage of individuals at a specific stage that progress to the next stage), sex ratio (proportion of female adults), and median adult survival age (age at which 50% of adults are still alive) were computed.

## Results

**Figure 2.**
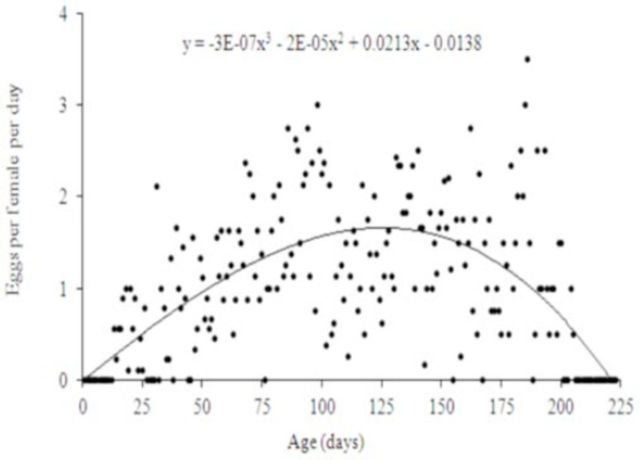
Age-specific oviposition rate of *Pseudotheraptus wayi* on French beans at 24.6 ± 1°C and 80 ± 1.3% RH. High quality figures are available online.

Table 1 shows the durations of various life stages and percentages of immatures surviving to subsequent stages. Total developmental duration (egg to adult emergence) was approximately 43 days. Sex ratio was 0.44. Fifty percent of females lived up to the age of 154 days, while the median adult survival age for males was 183 days. Mean fecundity was 171 ± 39.2 (ranging from 2–339) eggs per female. Daily egg production ranged from 0–14 eggs per female per day. Figure 2 shows mean daily egg production, with a polynomial relationship between number of eggs per female per day and age.

**Table 1.**
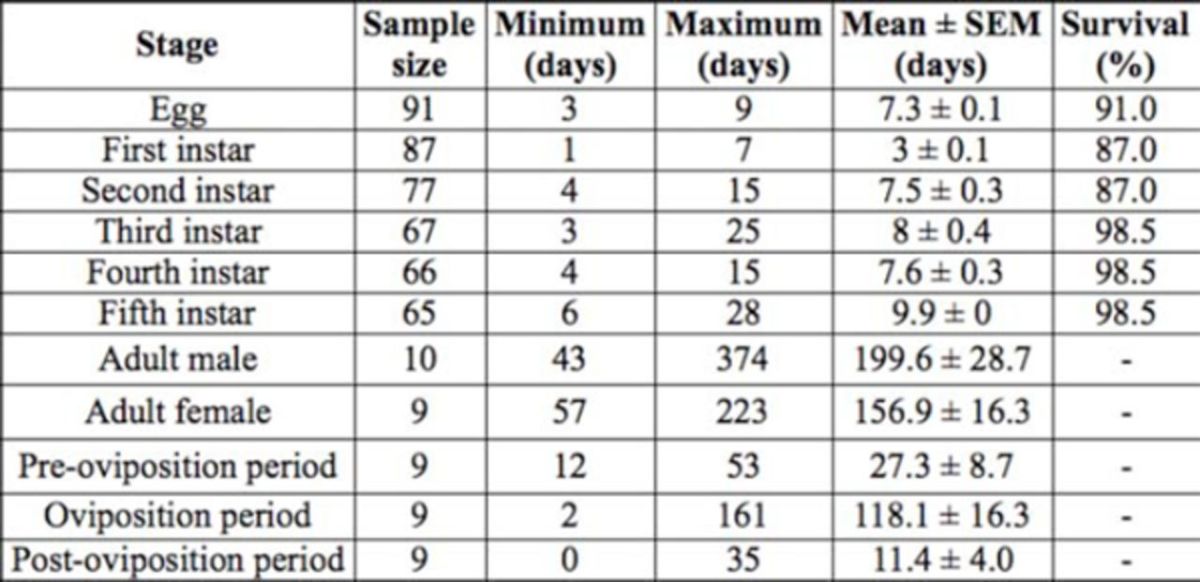
Durations of various life stages of *Pseudotheraptus wayi* and survival of immatures on French beans at 24.6 ± 1°C and 80 ± 1.3% RH.

## Discussion

Development of *P. wayi* from an egg to adult emergence on French beans compares favora-bly with development reported on coconut and cashew ( Way 1953 ; Wheatley 1961 ; Mitchell 2000 ; CABI 2005 ). Wheatley (1961) reported 10–15% mortality of first through second instars reared on coconut and described the mortality of later instars as very low, which corroborates our findings. This suggests that the first two instars are the most vulnerable stages and require more attention to minimize their mortality during mass rearing.

In the current study, males lived longer than females, which agrees with the findings of Way (1953) . Longevities of both sexes in the current study were however approximately thrice longer than those reported by Way (1953) , which indicates that French beans support a longer adult lifespan than coconut. Although the data from our study show a preoviposition period more than twice the range (9–13 days) reported in literature, indicating delayed sexual maturity on French beans, fecundity on French beans was almost twice that previously reported on coconut and cashew ( Way 1953 ; Mitchell 2000 ; CABI 2005 ). The maximum number of eggs laid per female per day on French beans was more than four times that which is reported on coconut and cashew ( Way 1953 ; Mitchell 2000 ). The trend of daily egg production indicates that the number of eggs laid by each female increases with age to a peak at ~100–150 days, then declines with age until the death of the female. The postoviposition period is so short, suggesting that most females reproduce throughout their life span once they attain sexual maturity.

This study is the first report of median adult survival ages for this species. This parameter was longer in males than females, probably because adult longevities were also longer in males than females. This parameter may be shorter on other hosts, such as coconut, which support shorter longevities of adult *P. wayi* .

In summary, based on adult longevity and fecundity in the current study, French bean pods seem to be a better food substrate for rearing *P. wayi* than coconut and cashew. The rearing technique described in this study may be useful for mass production of *P. wayi* for experimental purposes.
